# Evaluation of Serum Posaconazole Concentrations in Patients with Hematological Malignancies Receiving Posaconazole Suspension Compared to the Delayed-Release Tablet Formulation

**DOI:** 10.1155/2017/3460892

**Published:** 2017-06-11

**Authors:** Morgan Belling, Abraham S. Kanate, Alexandra Shillingburg, Xiaoxiao Lu, Sijin Wen, Nilay Shah, Michael Craig, Aaron Cumpston

**Affiliations:** ^1^Department of Pharmacy, West Virginia University Hospitals, Morgantown, WV, USA; ^2^Osborn Hematopoietic Malignancy and Transplantation Program, MBRCC, West Virginia University, Morgantown, WV, USA; ^3^Department of Biostatistics, West Virginia University, Morgantown, WV, USA

## Abstract

Posaconazole (PCZ) is frequently used for prophylaxis against invasive fungal infections (IFI) in patients undergoing induction chemotherapy for acute myeloid leukemia (AML) or myelodysplastic syndrome (MDS). Posaconazole is commercially available as an oral suspension (PCZ-susp) and as a delayed-release tablet (PCZ-tab). Differences in absorption and bioavailability between these formulations may result in variability in serum posaconazole concentrations. The primary objective of this retrospective analysis was to compare attainment of goal serum posaconazole steady state concentrations (*C*_ss_) ≥ 700 ng/ml in patients with AML/MDS undergoing induction chemotherapy receiving PCZ-susp 600–800 mg per day (*N* = 118) versus PCZ-Tablet 300 mg twice daily for one day, followed by 300 mg daily (*N* = 64). Sixty-two patients (97%) in the PCZ-tab group compared to 20 patients (17%) in the PCZ-susp group achieved goal *C*_ss_  (*P* < 0.0001). Median posaconazole serum *C*_ss_ was 1,665 ng/ml (522–3,830 mg/ml) in the PCZ-tab group versus 390 ng/ml (51–1,870 ng/ml) in the PCZ-susp group (*P* < 0.0001). There was no difference in hepatotoxicity, QTc prolongation, or breakthrough IFI. Patients receiving PCZ-tab were significantly more likely to achieve goal *C*_ss_ and demonstrated higher *C*_ss_ versus patients receiving PCZ-susp. Prospective studies are needed to assess the potential correlation of serum concentrations with efficacy and toxicity.

## 1. Introduction

Patients with acute myeloid leukemia (AML) or myelodysplastic syndrome (MDS) undergoing induction chemotherapy are severely immunocompromised and therefore at risk of developing invasive fungal infections (IFI). Posaconazole (PCZ) has the widest spectrum of antifungal activity among the triazole drug class and has demonstrated superiority versus fluconazole in the prevention of IFI in patients with AML or high-grade MDS undergoing induction chemotherapy and in patients with graft-versus-host disease (GVHD) receiving immunosuppression [1, 2]. Posaconazole is commercially available as an oral suspension and as a delayed-release tablet [3]. Substantial differences in absorption and bioavailability between these two formulations may result in variability in serum posaconazole concentrations. As demonstrated in several studies, bioavailability and gastrointestinal absorption of PCZ-susp are variable and affected by factors such as concomitant acid suppression therapy and enteral intake [4–9]. In contrast, PCZ-tab has demonstrated improved oral bioavailability. This formulation does not require food intake or a fatty meal to improve absorption and does not seem to be affected by use of acid suppressing agents [10–13]. These factors are especially important, considering the patient population being treated. Most patients receiving intensive chemotherapy and/or having GVHD are likely to have poor oral intake and be receiving stress ulcer prophylaxis.

Serum concentrations of posaconazole follow dose-dependent pharmacokinetics at steady state. Saturation of absorption of the PCZ-susp occurs at doses of 800 mg [14]. Serum concentrations of posaconazole correlate with clinical efficacy and inadequate concentrations are associated with breakthrough fungal infections [15–19]. Thus, differences in bioavailability between the two formulations may correspond to differences in serum concentrations and, subsequently, clinical effectiveness in preventing IFIs. Currently, guidelines recommend goal serum posaconazole concentrations ≥ 700 ng/ml for purposes of fungal prophylaxis [20]. Limited data is available comparing achievement of serum concentrations between the two formulations [21–24].

We conducted a retrospective analysis to compare attainment of goal serum posaconazole concentrations ≥ 700 ng/ml in patients with AML or high-grade MDL undergoing induction chemotherapy receiving the oral suspension versus the delayed-release tablets. We also assessed incidence and severity of toxicities, as well as rates of breakthrough IFIs. This study was approved by the Institutional Review Board at West Virginia University.

## 2. Patients and Methods

Patients with AML or high-grade MDS admitted to the inpatient hematologic malignancy service at West Virginia University Hospital between February 2008 and December 2015 were eligible. Posaconazole prophylaxis is standard of care at our institution for adult patients receiving systemic chemotherapy and expected to have prolonged neutropenia, defined as an expected nadir absolute neutrophil count of <500/*μ*l and duration of ≥7 to 10 days. Patients who were receiving concomitant medications interacting with posaconazole were excluded from the study. From February 2008 to December 2013, patients received PCZ-susp 600 to 800 mg per day. From January 2014 to December 2015, patients received PCZ-Tablet  300 mg twice daily on day 1, followed by 300 mg daily. Posaconazole concentrations were drawn after ≥7 days of therapy to permit adequate time to achieve steady state concentration (*C*_ss_). These concentrations were drawn approximately 4 hours after the posaconazole dose was administered in the morning. Only patients with a serum concentration obtained within these parameters were included for this analysis. Starting June 2014, for patients whose posaconazole concentration was ≥2,000 ng/ml, the dose was decreased from 300 mg daily to 200 mg daily to avoid toxicity without compromising adequate concentrations to be effective for prophylaxis. In August 2015, we began obtaining a second serum concentration after ≥7 days of reduced maintenance dose in order to assess the impact of dose reduction on serum concentrations.

Toxicities were analyzed according to the Common Terminology Criteria for Adverse Events (CTCAE) version 4.03 [25]. Grade 2 and higher toxicities were deemed clinically relevant. Specifically, incidence and severity of hepatotoxicity and QT_c_ prolongation were monitored, as these are relevant adverse effects of posaconazole therapy. Hepatotoxicity was assessed by obtaining liver function tests (LFTs) at least twice per week while patients were on posaconazole. QT_c_ prolongation was assessed at baseline and at physician discretion during therapy. Starting July 2015, an electrocardiogram (ECG) was consistently repeated while patients were receiving posaconazole prophylaxis at the time of serum concentration monitoring. Breakthrough fungal infections were classified as proven, probable, or possible according to commonly accepted criteria [26]. Clinical suspicion for fungal infections prompted assessment and work-up, including a chest CT scan and serum galactomannan and 1,3-*β*-D glucan assays. When possible, a bronchoalveolar lavage was performed and galactomannan was assessed on that sample.

Also assessed in this study were patient-specific factors that may impact serum posaconazole concentrations, including age, obesity, nausea, vomiting, diarrhea, mucositis, and concomitant acid suppression therapy [27]. Nutritional status was assessed by a registered dietician, who classified patients into three categories based on food intake. Category 1 included patients who consumed >75% of meals or >2 nutritional supplements per day. Category 2 included patients who consumed 50%–75% of meals or 1-2 nutritional supplements per day. Category 3 featured patients consuming <50% of meals or no nutritional supplements per day. All of these factors were assessed at the time the serum posaconazole concentration was drawn.

Descriptive statistics were utilized to assess patient characteristics. Fisher's exact test was used to assess independence between categorical variables, while the Wilcoxon rank sum test was used to assess differences between continuous variables.

## 3. Results

One-hundred and eighteen patients were included in the PCZ-susp group, while 64 patients were included in the PCZ-tab arm of the study. Most of the baseline patient characteristics were similar between the two groups (Table 1), except that more patients in the PCZ-susp group received high-dose cytarabine therapy compared to PCZ-tab group (13% versus 0%), whereas more patients in the PCZ-tab were noted to have better nutritional status.

**Figure 1:**
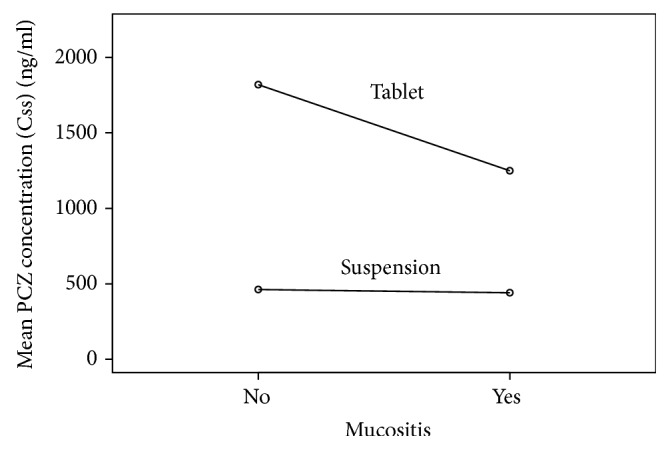
Linear regression model of posaconazole concentrations stratified by mucositis and treatment group. PCZ-tab group was significantly decreased by presence of mucositis, whereas no effect was seen in the PCZ-susp group.

In the PCZ-tab group 97%  (*n* = 62) achieved goal serum concentrations ≥ 700 ng/ml, while only 17%  (*n* = 20) reached goal concentrations in the PCZ-susp (*P* < 0.0001). The median *C*_ss_ in the PCZ-tab group was 1,665 (522–3,830) ng/ml versus 390 (51–1,870) ng/ml in the PCZ-susp cohort (*P* < 0.0001). Linear regression models (Table 2) revealed that age ≥ 60 years improved posaconazole concentrations, whereas mucositis and poor nutritional status (category 3) negatively impacted posaconazole concentrations. We also examined the influence of specific factors within each formulation (suspension or delayed-release tablet). In patients receiving PCZ-tab, those with mucositis had significantly decreased serum posaconazole concentrations compared to patients who did not have mucositis (*P* = 0.015; Figure 1). This effect was not seen in the PCZ-susp patients. Acid suppression therapy did not affect the posaconazole serum concentrations in either formulation group.

Grade ≥ 2 hepatotoxicity attributable to posaconazole was seen in 1 patient in each group. The serum posaconazole concentrations in these patients who developed hepatic dysfunction were 390 ng/ml (PCZ-susp) and 3,350 ng/ml (PCZ-tab). ECGs obtained while receiving posaconazole therapy were available for 32 and 38 patients in the PCZ-susp and PCZ-tab groups, respectively. Grade ≥ 2 QT_c_ prolongation occurred in 9%  (*n* = 3) receiving PCZ-susp compared to 21%  (*n* = 8) patients taking PCZ-tab (*P* = 0.21). Dose reduction of the maintenance posaconazole from 300 mg to 200 mg daily occurred in 14 patients. An additional posaconazole concentration obtained after ≥7 days of therapy with the adjusted dose in 4 of these patients noted a median concentration of 1,820 ng/ml (range, 954–2,790).

The incidence of breakthrough fungal infections was not statistically different between the two groups, with 8 cases (3 proven, 1 probable, and 4 possible) in the PCZ-susp arm and 4 cases (1 proven, 0 probable, and 3 possible) in the PCZ-tab group (*P* = 0.99). In the PCZ-susp arm, among the 3 proven cases of fungal infections, 2 were attributable to* Candida glabrata *(serum posaconazole concentrations 440 ng/ml and 700 ng/ml) and 1 to *Alternia* species (serum posaconazole concentration 341 ng/ml). In the PCZ-tab group, the proven infection was due to* Scedosporium species *(serum posaconazole concentration was 1,990 ng/ml).

## 4. Discussion

This is one of the largest studies comparing serum posaconazole concentrations with the suspension versus delayed-release tablet formulation. The rate of attainment of goal *C*_ss_ ≥ 700 ng/ml and a higher median *C*_ss_ were both significantly greater with PCZ-tab versus PCZ-susp, with no difference in the toxicity profile. Interestingly, the presence of mucositis was associated with a statistically significant decrease in posaconazole serum concentrations in the patients receiving PCZ-tab. However, all these patients attained the goal *C*_ss_ ≥ 700 ng/ml, thus likely negating any significant differences in clinical outcomes. No differences were noted in the incidence of hepatotoxicity or breakthrough fungal infections between groups.

The increased exposure of posaconazole tablets is thought to be a result of the tablet formulation's improved bioavailability and absorption. The PCZ-tab is formulated with a pH-dependent polymer that prevents the dissolution of the tablet in the acidic environment of the stomach. The subsequent increase in pH in the small intestine promotes dissolution of the PCZ-tab, resulting in improved bioavailability and absorption versus the PCZ-susp. Although a higher number of patients with better nutritional intake were seen in the PCZ-tab group, this should not have a substantial effect on the tablet absorption due to its delayed-release formulation. While no significant difference in rates of QT_c_ prolongation was noted between the two groups, the 21% incidence of QT_c_ prolongation seen in PCZ-tab may have clinical ramifications in a larger cohort. Of the 8 patients with documented QT_c_ prolongation in the PCZ-tab group, all had *C*_ss_ > 1,000 ng/ml, and 50% of these patients had *C*_ss_ > 2,000 ng/ml. Furthermore, all 8 of these concentrations (median 2,040 ng/ml) in the PCZ-tab group were higher than any of the 3 concentrations (median 426 ng/ml) associated with clinically significant QT_c_ prolongation in the PCZ-susp group. In the PCZ-susp group, the QTc prolongation noted in 8% of the patients may not be related to posaconazole since the ECGs were only ordered at physician discretion as part of the work-up for cardiac symptoms, whereas more PCZ-tab patients received routine ECG monitoring. A recently published phase III study assessing pharmacokinetics and safety of PCZ-tab in high-risk patients demonstrated no increase in the rate of adverse events with higher posaconazole exposure, but the QTc assessments in this cohort remain unclear [28]. Larger, prospective studies and postmarketing reporting are necessary to assess the association of QT_c_ prolongation with the higher posaconazole exposure with PCZ-tab and its clinical relevance.

We did not observe any differences in the rates of breakthrough fungal infections between the two groups, although the smaller sample size may have been inadequate to detect it. Previous, larger trials have documented that posaconazole exposure above goal concentrations correlates to a reduction in breakthrough IFIs. The potential protective effects of dose reducing the PCZ-tab patients to 200 mg in patients with higher serum concentrations are undetermined. Dose reductions may have improved the tolerability and toxicity profile of the PCZ-tab group. It is reassuring to note that dose reductions (*n* = 14) in PCZ-tab group did not affect serum concentrations adversely. Considering the smaller sample size and retrospective nature of our study, this needs to be further explored in a prospective manner.

Optimal timing of serum concentration monitoring is unclear and has varied in previous studies. The most commonly used time point is serum trough concentrations. For purposes of our analysis, we chose to evaluate 4-hour concentrations in our tablet patients, to remain consistent with our previous reports of the suspension formulation [9, 15] and allow direct comparison between the two groups. Considering the long half-life of posaconazole, we do not expect to see significant variations between these time points, but there is limited data to describe this variability.

In conclusion, our study showed more consistent attainment of goal posaconazole serum concentrations and superior median *C*_ss_ with PCZ-tab compared to PCZ-susp, thus supporting the routine use of the delayed-release tablet formulation as antifungal prophylaxis for patients undergoing induction chemotherapy for AML or high-risk MDS. The tolerability, safety, and effects of dose reduction of PCZ-tab seem acceptable but should be studied in prospective trials.

## Figures and Tables

**Table 1:** Patient characteristics.

Characteristic	Suspension patients (*N* = 118)	Tablet Patients (*N* = 64)	*P* value
Age (yr), median (range)	57 (18–84)	56 (20–75)	0.90

Male gender, *n* (%)	59 (50)	37 (58)	0.31

Weight (kg), median (range)	84 (38–175)	89 (59–194)	0.14

BMI^a^ (kg/m^2^), median (range)	30 (15–65)	29.1 (17–59)	0.91
BMI > 30 kg/m^2^, median (range)	58 (49)	30 (47)	0.77

Diagnosis, *n* (%)			
Acute myeloid leukemia	115 (97)	60 (94)	0.21
Myelodysplastic syndrome	3 (3)	4 (6)	

Chemotherapy regimen, *n* (%)			
Cytarabine-anthracycline (7 + 3)	80 (68)	48 (75)	0.016
High-dose cytarabine-based	15 (13)	0 (0)	
Clofarabine-based	7 (6)	5 (8)	
Mitoxantrone-etoposide	5 (4)	2 (3)	
Other	11 (9)	9 (14)	

Mucositis, *n* (%)	23 (19)	7 (11)	0.14

Diarrhea, *n* (%)	24 (20)	12 (19)	0.80

Vomiting, *n* (%)	14 (12)	7 (11)	0.85

Nutritional status^b^ *n* (%)			
Category 1	43 (36)	39 (61)	0.004
Category 2	41 (35)	16 (25)	
Category 3	18 (15)	8 (13)	
Unknown	16 (14)	1 (2)	

Acid suppression therapy, *n* (%)			
Proton pump inhibitor	44 (37)	21 (33)	0.18
H_2_ receptor antagonist	69 (58)	43 (67)	
None	5 (4)	0 (0)	

^a^BMI, body mass index. ^b^Nutritional status: Category 1: consumption of >75% of meals or >2 nutritional supplements per day; Category 2: consumption of 50%–75% or 1-2 nutritional supplements per day; Category 3: consumption of <50% of meals or no nutritional supplements per day.

**Table 2:** Linear regression models of the relation between PCZ serum concentration and treatment group and specific patient factors.

Characteristic	PCZ concentration effect^a^ (SE)	*P* value
PCZ treatment group		
Delayed-release tablet	1,294 (76)	<0.0001
Suspension	Reference	

Age ≥ 60 years	263 (118)	0.03

Male gender	41 (117)	0.73

BMI^b^ of >30 kg/m^2^	−69 (117)	0.55

Mucositis	−352 (157)	0.03

Diarrhea	−263 (147)	0.08

Vomiting	−140 (185)	0.45

Nutrition status		
Category 1	Reference	
Category 2	−205 (138)	0.14
Category 3	−417 (181)	0.02

Acid suppression therapy		
PPI	Reference	
H_2_ receptor antagonist	117 (122)	0.34
None	−526 (364)	0.15

^a^Effect, estimated mean difference; ^b^BMI, body mass index.
